# Supplementary material for: An outside individual option increases optimism and facilitates collaboration when groups form flexibly
Source: Nat Commun. 2024 Jun 29;15:5520. doi: 10.1038/s41467-024-49779-9 (PMC11217382; doi:10.1038/s41467-024-49779-9)
Supplement: Supplementary file 1 — Supplementary Information [file 41467_2024_49779_MOESM1_ESM.pdf]

Supplementary Information for

An outside individual option increases optimism and facilitates collaboration when groups form flexibly

Mori, R., Hanaki, N., & Kameda, T.*
*Corresponding author: tkameda@mi.meijigakuin.ac.jp

Supplementary Note 1

<u>Difference in Nash equilibrium predictions between mandatory and voluntary participation</u>

We first examine the threshold public goods game under mandatory participation (without the outside individual option). The game consists of five players who are each initially endowed with 10 points and can decide individually whether to cooperate (C; i.e., contribute their endowment, at a cost of 10 points) with the group or not (i.e., to defect, D; without the cost). If the number of players who contribute to the group reaches a certain threshold, q (here we consider only $q = \{2, 4, 5\}$), the public good is produced and all five players receive 30 extra points. However, if fewer than the threshold number of players contribute, the public good is not produced, and the contributions are not returned. Since our situation does not allow players to coordinate with one another, here we consider only a symmetrical Nash equilibrium.

Let us denote the probability of players' cooperation as p. As in Eq. 1 in the main text, the expected payoff, π, for each action (C or D) is computed as follows:

$$\begin{aligned} \pi_C(p, q) &= 30 \times \sum_{k=q-1}^{4} \binom{4}{k} p^k (1-p)^{4-k}, \\ \pi_D(p, q) &= 10 + 30 \times \sum_{k=q}^{4} \binom{4}{k} p^k (1-p)^{4-k} \end{aligned} \quad \text{(S1)}$$

Let us begin by searching for Nash equilibria consisting of pure strategies. The probability of players' cooperation p^* is a symmetrical pure-strategy Nash equilibrium if and only if the conditions $(p^* = 0 \text{ and } \pi_C(p^*, q) \leq \pi_D(p^*, q))$ or $(p^* = 1 \text{ and } \pi_C(p^*, q) \geq \pi_D(p^*, q))$ hold. Solving these equations, $p^* = 0$ when $q = 2, 4, 5$ and $p^* = 1$ when $q = 5$ are the only pure-strategy equilibria.

Next, let us look for Nash equilibria that involve mixed strategies ($0 < p^* < 1$). Now p^* must satisfy $\pi_C(p^*, q) - \pi_D(p^*, q) \equiv \Delta\pi = 0$. Further, for the equilibrium to be stable, slight deviations from the equilibrium need to be pushed back. Namely, a slight increase (decrease) in p from p^* should result in a negative (positive) Δπ, that is, $\frac{\partial \Delta\pi}{\partial p^*} < 0$. Solving these, we obtain $p^* \approx 0.41$ for $q = 2$, and $p^* \approx 0.88$ for $q = 4$.

To sum up, under mandatory participation, the threshold public goods game with each threshold has one stable noncooperative equilibrium where no one contributes ($p^* = 0$) and one stable cooperative equilibrium where a certain proportion of the population does contribute ($p^* > 0$). Importantly, the expected payoff, $E[\pi]$, for each equilibrium varies as follows:

Threshold (q)	Noncooperative equilibrium	Cooperative equilibrium
2	$(p^*, E[\pi]) = (0, 10)$	$(p^*, E[\pi]) = (0.41, 26.3)$
4	$(p^*, E[\pi]) = (0, 10)$	$(p^*, E[\pi]) = (0.88, 27.9)$
5	$(p^*, E[\pi]) = (0, 10)$	$(p^*, E[\pi]) = (1, 30)$

Now, we examine how the introduction of the outside individual option under voluntary participation alters the prediction. Recall that the outside individual option secures a more certain but less lucrative payoff compared to collaboration: Players choosing to leave (L) earn 20 points in total (10 extra points + 10 points for the initial endowment) regardless of other players' actions. Additionally, here we assume an infinite population and a nonzero probability of participation in

groups (we do not consider a situation where there are too few players who opt in to groups and even a single group of five members cannot be formed).

Notice that across the three thresholds, the expected payoff under the noncooperative equilibrium is less than the 20 points secured for the individual option (left with the initial endowment of 10 points), whereas the expected payoff under the cooperative equilibrium is greater than 20 points. As a result, players expecting the noncooperative equilibrium within groups should opt out of groups to receive the greater payoff of 20 points from the individual option. In other words, voluntary participation in our setting eliminates the noncooperative equilibrium and thereby facilitates group collaboration toward cooperation.

Supplementary Note 2

Additional tasks eliciting participants' economic and psychological characteristics

Apart from the main task (threshold public goods games), we included the following set of additional tasks and questionnaires to explore factors that possibly account for the individual heterogeneities in participants' play in the main task:

1. Other-regarding preference[1] (incentivized)
2. Risk preference[2] (incentivized)
3. Interpersonal Reactivity Index[3,4]
4. General trust[5]
5. Cognitive Reflection Test[6,7]
6. Intolerance of Uncertainty Scale[8]

First, to measure participants' other-regarding preferences, we used the inequity aversion model developed by Fehr and Schmidt (FS model[1]). The model presumes that a player's utility is determined by the weighted average of their own payoff and the inequity of payoff between themself and others, distinguishing between advantageous and disadvantageous inequity:

$$u_i = \pi_i - \alpha_i(\pi_j - \pi_i) - \beta_i(\pi_i - \pi_j) \qquad (S2)$$

where (π_i, π_j) denotes the payoff for the player and others, u_i is the resulting utility, and α_i and β_i are individual-level parameters that quantify the player's aversion to disadvantageous and advantageous inequity, respectively. As in He and Wu[9], participants are repeatedly asked to choose their preferred allocations of piles of money between themselves and another participant chosen at random (independently of the main task). On the basis of their choices, we estimated α and β for each participant.

Next, we presented participants with a gambling task to assess their risk attitudes: The participants were presented with a number of raffles with varying probabilities and prize amounts and were asked to choose one[2]. From their responses, we estimated participants' risk attitudes assuming constant relative risk aversion for their utility functions. Note that the Intolerance of Uncertainty Scale and the risk attitude measured with the gambling task differ not only methodologically (i.e., incentivized task vs. questionnaire) but also conceptually: The former is more concerned with attitudes toward the mere possibility of a negative event irrespective of the probability of its occurrence than toward known probabilities[8].

In both the allocation task and the gambling task, participants were directly incentivized to answer their preferences truthfully. Specifically, we randomly selected one target question from one target task and determined the participants' actual bonus according to the exact procedure

specified (e.g., dividing a pile of money with another randomly selected participant or holding the raffle they selected). Please refer to a separate file located at https://github.com/ryutau/voluntary-collaboration/blob/main/documents/main_experiment_materials.pdf for the complete set of task items.

We exploratively analyzed the relationships between these participants' economic and psychological characteristics and their individualistic choices in the threshold public goods game. As in previous research reporting little bearing of risk preference or distributive preference[10,11] (as measured by the social value orientation slider[12]), our results also showed that neither risk aversion nor other-regarding preference (as measured with the FS model[1]) predicted participants' choice of the individual option. See Supplementary Table 1 for the summary.

Supplementary Note 3

Comparison of raw expectations and pivotal probabilities in predicting cooperation within groups

In the threshold public goods game, players gave their own actions as well as their expectations about other players' actions ("How likely are others to cooperate?"). How did their expectations determine their actions? Standard theories of expected utility maximization dictate that players should not respond to their raw expectations but to the pivotal probability of their own decisions (i.e., both necessary and sufficient for the provision of collective benefits) calculated from them: Players expecting higher pivotal probabilities are more likely to cooperate. However, we found that raw expectations were a better predictor of their cooperation: Players who expected other players to cooperate more were more likely to cooperate themselves.

Calculating the difference in the areas under the curves of the receiver operating characteristic (ROC-AUC) in predicting cooperation, we found they were significantly greater than zero across conditions ($\Delta\mathrm{AUC} \equiv \mathrm{AUC}_{\mathrm{raw\ expectation}} - \mathrm{AUC}_{\mathrm{pivotal\ probability}}$: $q = 2$, mandatory: $\Delta\mathrm{AUC} = 0.52$, 95% CI $[0.39, 0.65]$; $q = 2$, voluntary: $\Delta\mathrm{AUC} = 0.49$, 95% CI $[0.30, 0.66]$; $q = 4$, mandatory: $\Delta\mathrm{AUC} = 0.13$, 95% CI $[0.07, 0.20]$; $q = 4$, voluntary: $\Delta\mathrm{AUC} = 0.36$, 95% CI $[-0.05, 0.63]$; when $q = 5$, the difference is 0 by definition). All CIs refer to bootstrapped 95% CIs. See also Supplementary Fig. 3 for illustration.

Supplementary Note 4

Model extension: Analyses of best responses under mandatory and voluntary participation

The details of our theoretical analyses are described in the main text. Here, we aim to strengthen the claim by showing some generality of the results in three ways. First, we show, through best-response analyses, that the two results of best-response analyses in the main text—two separable motives for defection in the mandatory threshold public goods game and self-selection of optimistic players into groups in the voluntary game—hold with other group sizes and threshold values. Then, concerning the impact of self-selection on the resultant cooperation rate under voluntary participation, we demonstrate that our investigation with beta distributions includes a wide variety of distributions for $\phi(\gamma)$ and also investigate if the results are robust against the changes in loners' payoff.

Best-response analyses. We examine the best response as a function of players' subjective beliefs about other players' cooperation in the threshold public goods game. Following the main text, we denote a player's belief about others' cooperativeness as $\gamma \in [0,1]$, their action as $x \in \chi$, and the resultant payoff as π. Here, we further denote the group size as N, the threshold value as q $(1 < q < N)$, the endowment as e (in points), the cost for cooperation as c (in points), the additional benefit from successful collaboration as b (in points), and the additional benefit of being a loner as r (in points). In this setup, the expected payoff of each action as a function of γ is

$$\begin{aligned} E[\pi|x = \mathrm{C}] &= e - c + b \times \Gamma_{q-1}, \\ E[\pi|x = \mathrm{D}] &= e + b \times \Gamma_{q}, \\ E[\pi|x = \mathrm{L}] &= e + r, \end{aligned} \tag{S3}$$

where L stands for leave and Γ_k denotes the probability that at least k of $N-1$ other members will cooperate: $\Gamma_k = \sum_{j=k}^{N-1} \binom{N-1}{j} \gamma^j (1-\gamma)^{(N-1)-j}$.

Let us first consider the mandatory game. The change points of gamma in terms of the corresponding best response action are the solutions of an equation:

$$\begin{gathered} E[\pi|x = \mathrm{C}] = E[\pi|x = \mathrm{D}] \\ \Leftrightarrow -c + bP_{\mathrm{piv}} = 0, \text{where } P_{\mathrm{piv}} = \binom{N-1}{q-1} \gamma^{q-1}(1-\gamma)^{N-q}. \end{gathered} \tag{S4}$$

P_{piv} corresponds to the probability that exactly $q-1$ members among the $N-1$ members other than oneself choose to cooperate. This probability is often called "pivotal probability" in threshold public game literature because it is the situation where the player's own cooperation is both sufficient and necessary for the group success.

Notice that $P_{\mathrm{piv}} = 0$ at both endpoints ($\gamma = 0, 1$) and $P_{\mathrm{piv}} > 0$ inward ($0 < \gamma < 1$), and an equation $\frac{\partial}{\partial \gamma} P_{\mathrm{piv}} = 0 \Leftrightarrow \mathrm{C}\gamma^{q-2}(1-\gamma)^{N-q-1} \times \{(q-1) - (N-1)\gamma\} = 0$, where C is some constant not depending on γ, has only one interior solution $\gamma = \frac{q-1}{N-1}$. Thus, P_{piv} takes its maximum at $\gamma = \frac{q-1}{N-1}$. Accordingly, as long as $P_{\mathrm{piv}|\gamma=\frac{q-1}{N-1}} > \frac{c}{b}$, Eq. S4 is satisfied with two values of γ, meaning that the conclusion of two separable motives for defection holds. When $P_{\mathrm{piv}|\gamma=\frac{q-1}{N-1}} < \frac{c}{b}$, the added value of cooperation coming from the pivotal probability never exceeds the cost of cooperation, leading to no solutions of Eq. S4. This is the scenario in which the model based on expected payoff maximizing predicts complete defection among players (cf. voting paradox).

Under voluntary participation, whether a player participates in a group or not is determined by the comparison between the expected payoff of being a loner ($E[\pi|\mathrm{L}]$) and the maximum expected payoff within a group ($\max(E[\pi|\mathrm{C}], E[\pi|\mathrm{D}])$). As $E[\pi|\mathrm{L}]$ is constant regardless of γ, and $\max(E[\pi|\mathrm{C}], E[\pi|\mathrm{D}])$ increases as γ increases, the change point at which the best response shifts from leaving to participating in groups occurs at most once. Thus, it follows that players naturally opt to be a loner unless they hold sufficiently high expectations about others cooperating (γ), assuming reasonable values for the benefit of staying out (r).

Impact of self-selection on cooperation rate. Next, we examine the impact of self-selection on the resultant cooperation rate within groups (among those who opt for groups). As detailed in the

main text, assuming the population-level distribution of beliefs, $\phi(\gamma)$, the resultant cooperation rate is obtained as

$$p_{\mathrm{coop}} = \frac{r_{\mathrm{C}}}{r_{\mathrm{C}} + r_{\mathrm{D}}}, \text{where } r_x = \int_0^1 I\{\text{Best response is } x, \text{given } \gamma\}\, \phi(\gamma)\, \mathrm{d}\gamma\,. \tag{S5}$$

Since the result should vary depending on $\phi(\gamma)$, we numerically explored how the cooperation rate (p_{coop}) differs between mandatory and voluntary participation while systematically altering the shape of $\phi(\gamma)$. Specifically, we employed the Beta distribution, $\mathrm{Beta}(a, b)$. By adjusting both a and b from 1 to 15, we generated a broad spectrum of distributions. Refer to Supplementary Fig. 4 for an illustration.

Supplementary Fig. 1 shows the comparison of resultant cooperation rates under mandatory and voluntary (i.e., with the leaving option) participation, fixing payoff parameters ($e = c = 10, b = 30, r = 10$), and group size ($N = 5$). Here we extend the analyses in terms of a loner's payoff, r. Supplementary Fig. 5 shows the changes in cooperation rate from mandatory to voluntary participation: $p_{\mathrm{coop,voluntary}} - p_{\mathrm{coop,mandatory}}$, as a function of $\phi(\gamma)$. The cell colors represent increases (orange), decreases (blue), and no changes (white) in cooperation rate on a continuous scale. Each row corresponds to a different value of the additional payoff for loners, r, ranging from 5 to 15 (in increments of 2.5). The result suggests that the positive impact of self-selection under voluntary participation holds even when the payoff for loners is decreased. Increasing r amplifies the caveats mentioned earlier, as a more attractive outside option diminishes the relative appeal of participating in a group. Those who are to leave the group first would be those with a lower expected payoff within the group, who should thus be more likely to be cooperators trying to contribute to successful collaborations rather than free riders trying to benefit from them without incurring private costs.

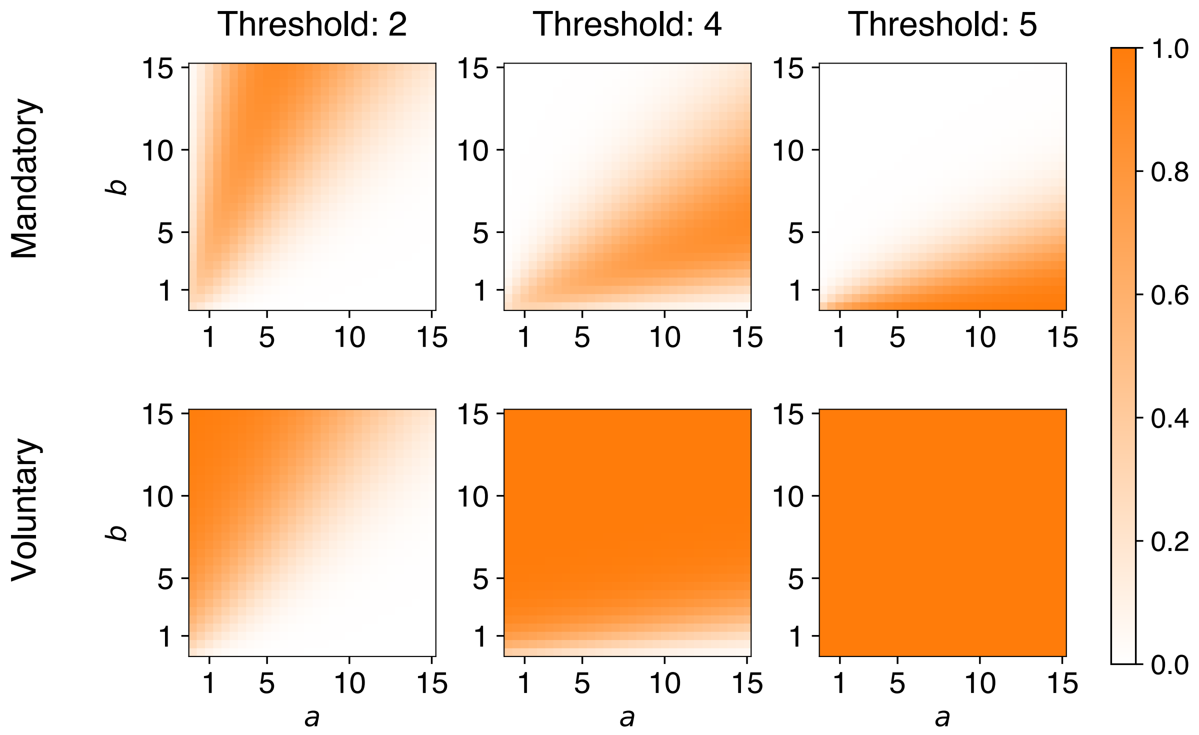

Supplementary Fig. 1.

Voluntary cooperation induces higher cooperation rates via self-selection across a wide range of parameters of the Beta distribution. Resultant rates of cooperation (see Eqs. 2 and 3 in the main text) as a function of the distribution of beliefs, $\phi(\gamma)$. We assume that $\phi(\gamma) \sim \mathrm{Beta}(a, b)$. The deeper orange indicates that the cooperation rate is closer to 1 and the lighter color shows the cooperation rate closer to 0. The panels correspond to the six conditions in Fig. 3C and are implemented in the experiment. By comparing the results within each threshold value, we can confirm that voluntary participation induces higher cooperation rates compared to mandatory participation across a wide range of parameters.

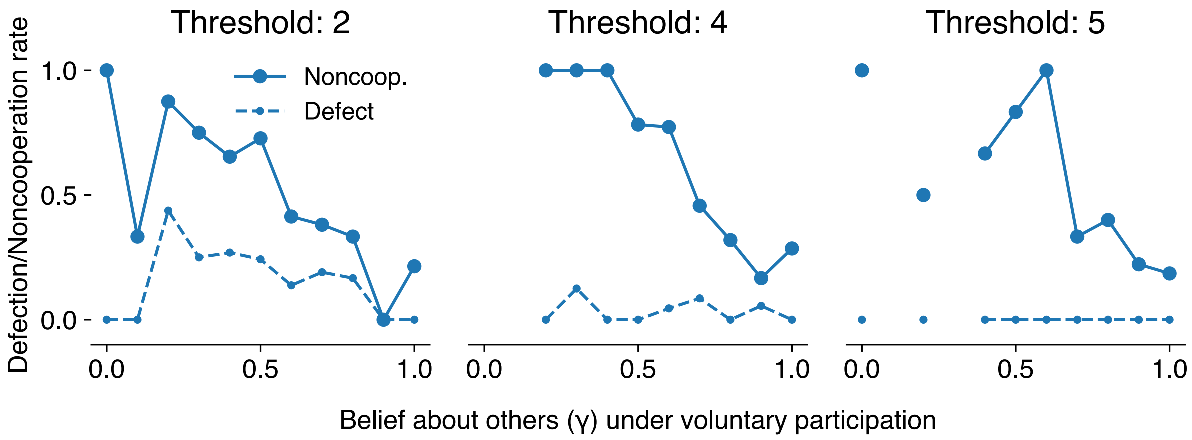

Supplementary Fig. 2. Defection rates and noncooperation rates as a function of participants' beliefs about others' cooperativeness in the voluntary conditions ($n = 191$ individuals). Regardless of the thresholds, individual defection rates and noncooperation (defection + leaving) rates decreased almost monotonically as participants' expectations about others' cooperativeness increased. Recall that the same pattern was also observed in the mandatory conditions (Fig. 3A in the main text). Note that when the threshold was 5, the defection rate (dashed lines) remained zero as there was no defector in this condition.

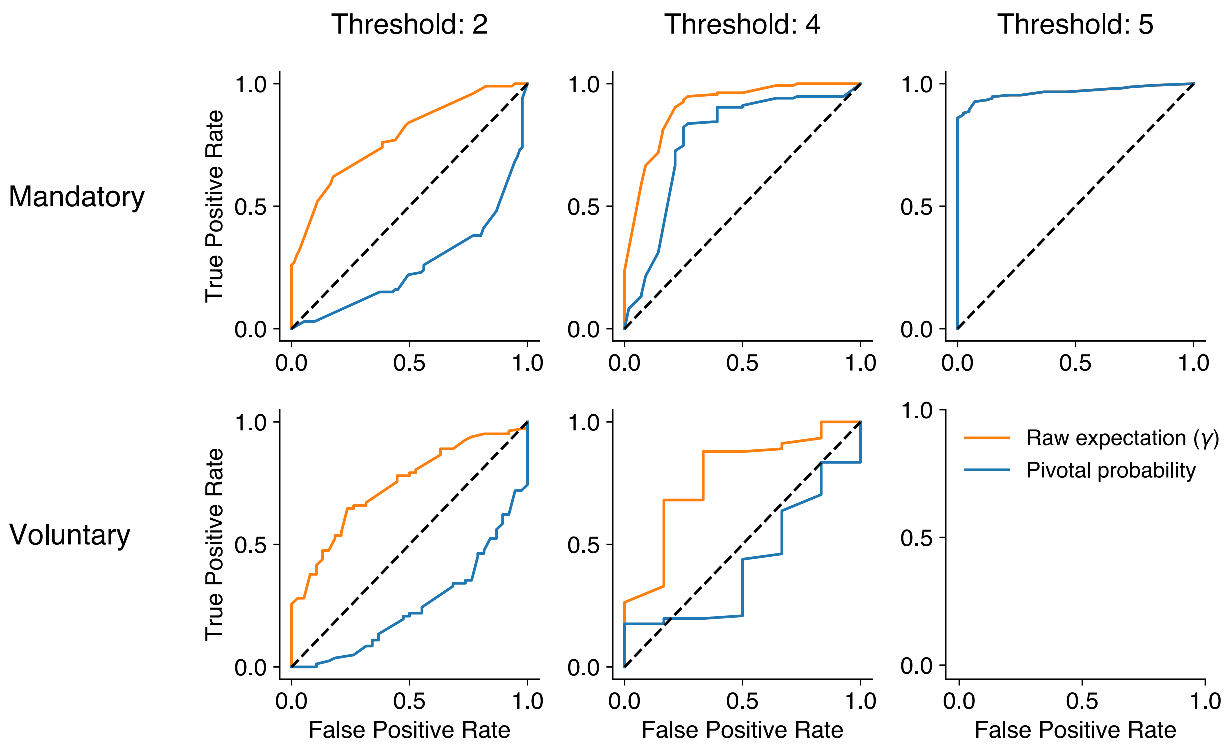

Supplementary Fig. 3. Comparison of ROC curves of participants' raw expectations and pivotal probabilities in predicting their cooperation decisions. We compared the goodness of each prediction by analyzing the respective receiver operating characteristic (ROC) curve used in the signal detection framework. The more the curve is situated above the diagonal, the better the prediction. Results indicate that the raw expectation predicts participants' cooperation (orange curves) better than the pivotal probability (blue curves) regardless of the condition, with the exception of when the threshold is 5, where both values have the same order, resulting in identical ROC curves (thus the two curves completely overlap, though displayed only in blue, in the upper-right panel). Observe that when the threshold is 2, the ROC curves drawn from the pivotal probability (blue curves) consistently lie below the diagonal, indicating the predictions were even inferior to completely random predictions. The statistical analysis in the supplementary text is based on the comparison of the area under the curve (AOC) of the two curves—the greater the AOC, the better the prediction. The lower right panel (when the threshold value is 5 and participation is voluntary) is empty because there were no defectors in this condition.

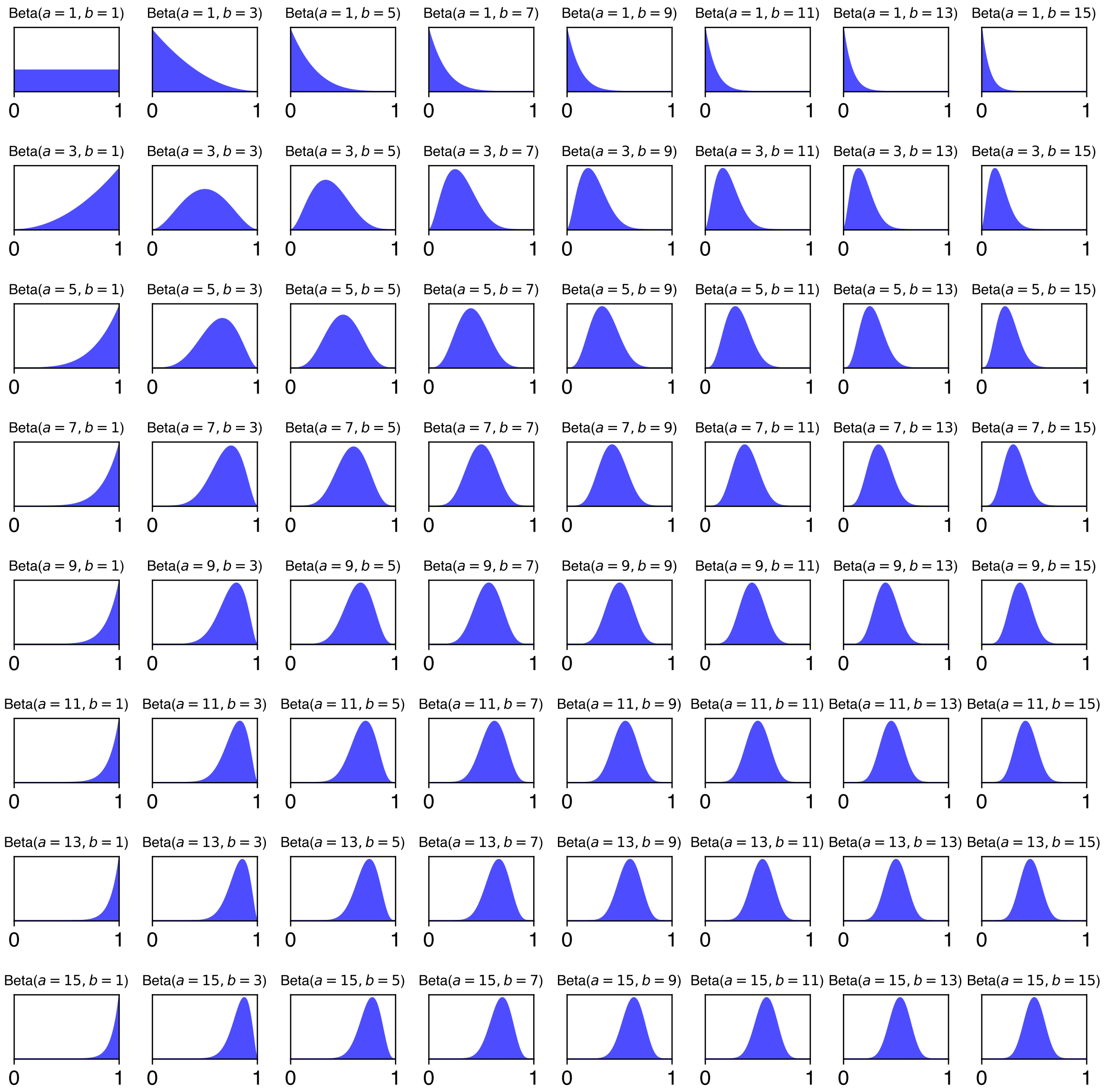

Supplementary Fig. 4. Illustration of the breadth distributions investigated for $\phi(\gamma)$. We employed the Beta distribution, manipulating its parameters within the ranges of $1 \le a, b \le 15$. This approach yielded a diverse array of distributions, showcasing significant variations.

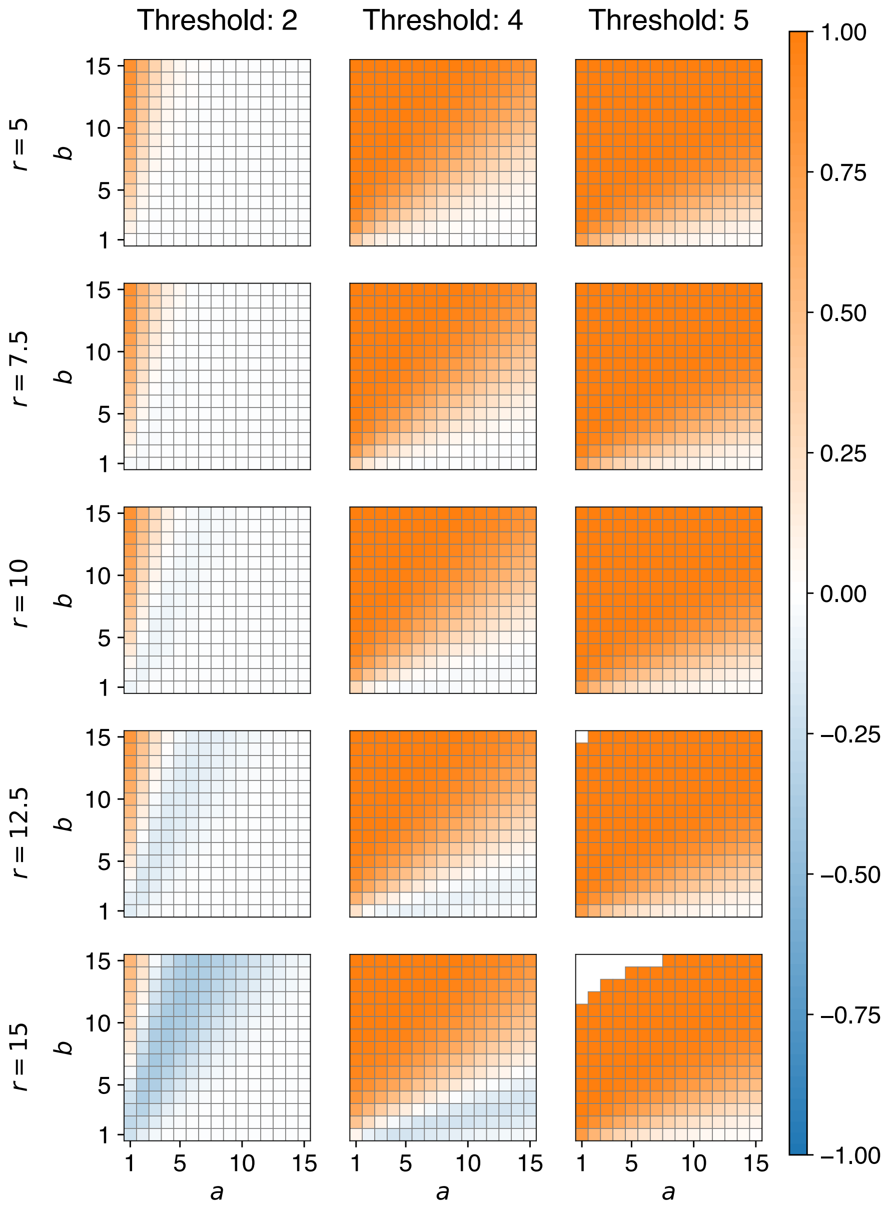

Supplementary Fig. 5. Extended results about the impact of self-selection on the cooperation rate within groups. The figure shows the resultant cooperation rates as a function of the distribution of beliefs, $\phi(\gamma) \sim \text{Beta}(a, b)$. Most parameters are set following the main text: $N = 5, e = c = 10, b = 30$. Each column of panels corresponds to a different threshold value ($q \in \{2,4,5\}$) and each row of panels corresponds to a different payoff for loners ($r = \{5,\ 7.5,\ 10,\ 12.5,\ 15\}$). Within each panel, the position of a cell corresponds to a specific shape for $\phi(\gamma)$, where the *x* and *y* axes indicate parameters a and b, respectively. The cell color represents the changes in cooperation rate by the introduction of self-selection (i.e., from mandatory to voluntary participation); $p_{\text{coop,voluntary}} - p_{\text{coop,mandatory}}$. Orange indicates the cooperation rate increases under voluntary participation, whereas blue indicates the opposite, and white indicates no change. Note that some cells lack gray frames, denoting missing values that occur because the denominator in the cooperation rate equation (Eq. S4) approaches zero owing to the effects of truncation in numerical computations.

Supplementary Table 1. Estimated parameters of the mixed-effects logistic regression predicting the decisions to choose the individual option

Variable	z value	Estimate	p value	95 percentile
(Intercept)	-1.28	-0.42	0.18	[-1.09, 0.18]
Disadvantageous aversion (α in FS model)	1.46	0.23	0.09	[-0.03, 0.59]
Advantageous aversion (β in FS model)	-1.36	-0.11	0.15	[-0.28, 0.04]
Risk aversion	0.28	0.03	0.74	[-0.18, 0.26]
Empathic concern (in the IRI)	-0.58	-0.07	0.55	[-0.33, 0.16]
Fantasy (in the IRI)	0.85	0.11	0.39	[-0.11, 0.36]
Personal distress (in the IRI)	-1.00	-0.13	0.33	[-0.36, 0.14]
Perspective taking (in the IRI)	1.59	0.20	0.10	[-0.04, 0.46]
Intolerance of uncertainty	1.87	0.23	0.07	[-0.02, 0.47]
Cognitive reflection test score	0.50	0.06	0.65	[-0.18, 0.31]
General trust	-1.43	-0.19	0.16	[-0.45, 0.08]
Age	-1.64	-0.22	0.05	[-0.55, -0.01]
Gender (male)	1.04	0.24	0.29	[-0.18, 0.70]
Gender (other)	-0.20	-0.35	0.55	[-1.95, 0.83]
Threshold	-0.69	-0.05	0.48	[-0.20, 0.09]

Note. Independent variables include the threshold value and participants' demographic data as well as economic, psychological, and cognitive characteristics that were measured separately from the main task. All numerical variables, except threshold values, are standardized across participants to have a mean of 0 and a standard deviation of 1; thus, the estimates shown are standardized coefficients. FS = Fehr and Schmidt model[1]. IRI = Interpersonal Reactivity Index[3].

Supplementary References

1. Fehr, E. & Schmidt, K. M. A Theory of fairness, competition, and cooperation. *Q. J. Econ.* **114**, 817–868 (1999).
2. Holt, C. A. & Laury, S. K. Risk aversion and incentive effects. *Am. Econ. Rev.* **92**, 1644–1655 (2002).
3. Davis, M. H. A multidimensional approach to individual differences in empathy. *JSAS Catalog of Selected Documents in Psychology* **10**, 85 (1980).
4. Himichi, T. et al. Development of a Japanese version of the Interpersonal Reactivity Index. *Shinrigaku Kenkyu* **88**, 61–71 (2017).
5. Yamagishi, T. & Yamagishi, M. Trust and commitment in the United States and Japan. *Motiv. Emot.* **18**, 129–166 (1994).
6. Toplak, M. E., West, R. F. & Stanovich, K. E. Assessing miserly information processing: An expansion of the Cognitive Reflection Test. *Think. Reason.* **20**, 147–168 (2014).
7. Baron, J., Scott, S., Fincher, K. & Emlen Metz, S. Why does the Cognitive Reflection Test (sometimes) predict utilitarian moral judgment (and other things)? *J. Appl. Res. Mem. Cogn.* **4**, 265–284 (2015).
8. Carleton, R. N., Norton, M. A. P. J. & Asmundson, G. J. G. Fearing the unknown: A short version of the Intolerance of Uncertainty Scale. *J. Anxiety Disord.* **21**, 105–117 (2007).
9. He, H. & Wu, K. Choice set, relative income, and inequity aversion: An experimental investigation. *J. Econ. Psychol.* **54**, 177–193 (2016).
10. Gross, J. & De Dreu, C. K. W. Individual solutions to shared problems create a modern tragedy of the commons. *Sci. Adv.* **5**, eaau7296 (2019).
11. Gross, J. & Böhm, R. Voluntary restrictions on self-reliance increase cooperation and mitigate wealth inequality. *Proc. Natl. Acad. Sci. U. S. A.* **117**, 29202–29211 (2020).
12. Murphy, R. O., Ackermann, K. A. & Handgraaf, M. Measuring social value orientation. *SSRN Electronic J.* **21**, 875–903 (2011).
