# Supplementary material for: An outside individual option increases optimism and facilitates collaboration when groups form flexibly
Source: Nat Commun. 2024 Jun 29;15:5520. doi: 10.1038/s41467-024-49779-9 (PMC11217382; doi:10.1038/s41467-024-49779-9)
Supplement: Supplementary file 3 — Reporting Summary [file 41467_2024_49779_MOESM3_ESM.pdf]

nature portfolio

Corresponding author(s): Tatsuya Kameda

Last updated by author(s): Jun 4, 2024

Reporting Summary

Nature Portfolio wishes to improve the reproducibility of the work that we publish. This form provides structure for consistency and transparency in reporting. For further information on Nature Portfolio policies, see our Editorial Policies and the Editorial Policy Checklist.

Statistics

For all statistical analyses, confirm that the following items are present in the figure legend, table legend, main text, or Methods section.

n/a	Confirmed	
☐	☒	The exact sample size (*n*) for each experimental group/condition, given as a discrete number and unit of measurement
☐	☒	A statement on whether measurements were taken from distinct samples or whether the same sample was measured repeatedly
☐	☒	The statistical test(s) used AND whether they are one- or two-sided<br>*Only common tests should be described solely by name; describe more complex techniques in the Methods section.*
☐	☒	A description of all covariates tested
☒	☐	A description of any assumptions or corrections, such as tests of normality and adjustment for multiple comparisons
☐	☒	A full description of the statistical parameters including central tendency (e.g. means) or other basic estimates (e.g. regression coefficient) AND variation (e.g. standard deviation) or associated estimates of uncertainty (e.g. confidence intervals)
☐	☒	For null hypothesis testing, the test statistic (e.g. *F*, *t*, *r*) with confidence intervals, effect sizes, degrees of freedom and *P* value noted<br>*Give P values as exact values whenever suitable.*
☒	☐	For Bayesian analysis, information on the choice of priors and Markov chain Monte Carlo settings
☒	☐	For hierarchical and complex designs, identification of the appropriate level for tests and full reporting of outcomes
☒	☐	Estimates of effect sizes (e.g. Cohen's *d*, Pearson's *r*), indicating how they were calculated

Our web collection on statistics for biologists contains articles on many of the points above.

Software and code

Policy information about availability of computer code

Data collection	Both experiments were programmed in oTree (version 5.0.0), which is a Python-based library. Screen captures of instruction slides (translated to English) presented to participants in the experiments are publicly available in a github repository (DOI: 10.5281/zenodo.11427517).
Data analysis	The code used in this study is available at the GitHub repository: https://github.com/ryutau/voluntary-collaboration (DOI: 10.5281/zenodo.11427517). The analyses were implemented in Python (v.3.10.11) using the matplotlib (v.3.7.2) library, numpy (v.1.24.4), pandas (v.2.0.3), pymer4 (v.0.8.0), scikit-learn (v.1.3.0), and scipy (v.1.12.0).

For manuscripts utilizing custom algorithms or software that are central to the research but not yet described in published literature, software must be made available to editors and reviewers. We strongly encourage code deposition in a community repository (e.g. GitHub). See the Nature Portfolio guidelines for submitting code & software for further information.

Data

Policy information about availability of data

All manuscripts must include a data availability statement. This statement should provide the following information, where applicable:

- Accession codes, unique identifiers, or web links for publicly available datasets
- A description of any restrictions on data availability
- For clinical datasets or third party data, please ensure that the statement adheres to our policy

The data of our experiments are publicly available at https://github.com/ryutau/voluntary-collaboration. There are no restrictions to accessing the data.

Research involving human participants, their data, or biological material

Policy information about studies with human participants or human data. See also policy information about sex, gender (identity/presentation), and sexual orientation and race, ethnicity and racism.

Reporting on sex and gender	In the main experiment, according to their self-reported gender, 52% of our participants were female, 45% were male, one participant declared "other", and four participants chose not to answer. In the additional experiment, 38% of participants were female, 60% were male, and two participants chose not to answer. We did not perform any major analyses on gender, since we did not have reasons to believe that it would impact the results. We performed an exploratory analysis investigating the relationships between participants' demographic characteristics including their gender (plus psychological characteristics) with their individualistic choices in the threshold public goods game; This analysis yielded no significant results.
Reporting on race, ethnicity, or other socially relevant groupings	We did not collect data on race or ethnicity.
Population characteristics	Main experiment:Participants were between 19 and 47 years of age (M = 22.8, SD = 2.7); Additional experiment:Participants were between 18 and 35 years of age (M = 22.9, SD = 2.8).
Recruitment	Participants were recruited from the student population of the University of Tokyo (Tokyo, Japan) and Meiji Gakuin University (Tokyo, Japan) for the main experiment, and from the subject pool of the Institute of Social and Economic Research (ISER) at Osaka University for the additional experiment, both via online recruitment platforms.
Ethics oversight	The main experiment was approved by the Ethics Committee of the University of Tokyo (UTSP-21015), and the additional experiment was approved by the Ethics Committee of the ISER (No. 20240102).

Note that full information on the approval of the study protocol must also be provided in the manuscript.

Field-specific reporting

Please select the one below that is the best fit for your research. If you are not sure, read the appropriate sections before making your selection.

☐ Life sciences ☒ Behavioural & social sciences ☐ Ecological, evolutionary & environmental sciences

For a reference copy of the document with all sections, see nature.com/documents/nr-reporting-summary-flat.pdf

Behavioural & social sciences study design

All studies must disclose on these points even when the disclosure is negative.

Study description	We investigated how the availability of an outside individualistic alternative impacts group collaboration in human groups. In the theory development, we modeled group collaborative situations as people's voluntary choices between joining an uncertain public goods provisioning in groups and pursuing a certain but less profitable individual option. In two experiments, we employed a threshold public game with the outside individual option to empirically test our predictions The experimental data collected was quantitative.
Research sample	Our sample consists of students. In the main experiment, there were 191 participants (Gender: 86 male, 100 female, 1 other, and 4 chose not to answer; Age: M = 22.8, SD = 2.7). In the additional experiment, there were 182 participants (Gender: 110 male, 70 female, and 2 chose not to answer; Age: M = 22.9, SD = 2.8).<br>While the lack of representativeness in student samples should be considered when generalizing our findings, our rationale for using a student sample was to prioritize control in the online experiment. We used Zoom instructions to ensure participants' engagement and commitment to the experiment. Although samples from crowdsourcing platforms (e.g., Prolific) could be more representative of the general population, such platforms pose considerable technical challenges for conducting Zoom-based sessions as implemented in this study.
Sampling strategy	In the main experiment, we employed a 2 (group participation: mandatory or voluntary) × 3 (threshold: minimum number of cooperators required for group success in the public goods game) within-subject design. We ran six experimental sessions, each with approximately 30 participants (ranging from 30 to 35) taking part simultaneously. The game design required a minimum of 30 participants per session, and six sessions were needed to properly randomize the order of conditions. Power analysis based on a pilot experiment with the identical design indicated that a sample size of 180 should be sufficient to detect significant differences in cooperation rates between mandatory and voluntary group participation for our primary analysis. Therefore, we capped registration at a maximum of 35 individuals per session, resulting in 206 signups.<br>In the additional experiment, we had 3 within-subject conditions. We had 6 experimental sessions, each with around 30 participants (ranging from 28 to 32). To match the sample size with that of the main experiment, we capped registration at a maximum of 34 individuals per session, resulting in 204 signups.
Data collection	The experiments were programmed in oTree (version 5.0.0), which is Python (version 3.10.11) based. Participants received a link to the experiment website, and accessed the experiments from home while being connected simultaneously via Zoom.
Timing	Data were collected between 2022-02-02 and 2022-02-04 for the main experiment, and between 2024-01-29 and 2024-02-02 for the

additional experiment.

Data exclusions	Main experiment: All the 192 participants who showed up for the experiment completed the whole experiment. However, one participant afterward declared that she had already graduated from the university. This participant was excluded before analyzing the data, resulting in a total of 191 participants.<br>Additional experiment: All the 181 participant who showed up for the experiment completed the whole experiment.
Non-participation	Main experiment: Of 206 people who signed up, fourteen showed up late and failed to participate in the experiment.<br>Additional experiment: Of 204 people who signed up, twenty-one failed to participate in the experiment.
Randomization	Main experiment: There were a total of six conditions for the main task, using a 2 (Group Participation: mandatory or voluntary) × 3 (Threshold Value: 2, 4, or 5) factorial within-subject design. Each participant underwent all six conditions once. The order of the six conditions was (partially) randomized across participants: Half of the participants played all three voluntary conditions first while the other half played all three mandatory conditions first, with the order of 3 threshold values within the voluntary and mandatory conditions being randomized.<br>Additional experiment: There were a total of three within-subject conditions. The order of the conditions was fully randomized across participants.

Reporting for specific materials, systems and methods

We require information from authors about some types of materials, experimental systems and methods used in many studies. Here, indicate whether each material, system or method listed is relevant to your study. If you are not sure if a list item applies to your research, read the appropriate section before selecting a response.

Materials & experimental systems

n/a	Involved in the study
☒	☐ Antibodies
☒	☐ Eukaryotic cell lines
☒	☐ Palaeontology and archaeology
☒	☐ Animals and other organisms
☒	☐ Clinical data
☒	☐ Dual use research of concern
☒	☐ Plants

Methods

n/a	Involved in the study
☒	☐ ChIP-seq
☒	☐ Flow cytometry
☒	☐ MRI-based neuroimaging

Plants

Seed stocks	NA
Novel plant genotypes	NA
Authentication	NA
